# Diversity of Growth Responses of Soil Saprobic Fungi to Recurring Heat Events

**DOI:** 10.3389/fmicb.2020.01326

**Published:** 2020-06-19

**Authors:** Aleksandra Szymczak, Masahiro Ryo, Julien Roy, Matthias C. Rillig

**Affiliations:** ^1^Institute of Biology, Freie Universität Berlin, Berlin, Germany; ^2^Berlin-Brandenburg Institute of Advanced Biodiversity Research, Berlin, Germany

**Keywords:** thermal stress, soil saprobic fungi, global change, climate extreme, multiple perturbation events, stress priming

## Abstract

As a consequence of ongoing climate change, the frequency of extreme heat events is expected to increase. Recurring heat pulses may disrupt functions supported by soil microorganisms, thus affecting the entire ecosystem. However, most perturbation experiments only test effects of single heat events, and therefore it remains largely unknown how soil microorganisms react to repeated pulse events. Here we present data from a lab experiment exposing 32 filamentous fungi, originally isolated from the same soil, to sequential heat perturbations. Soil saprobic fungi isolates were exposed to one or two heat pulses: mild (35°C/2 h), strong (45°C/1 h), or both in sequence (35°C/2 h+45°C/1 h), and we assessed growth rate. Out of the 32 isolates 13 isolates showed an antagonistic response, 3 isolates a synergistic response and 16 isolates responded in an additive manner. Thus the 32 filamentous fungal isolates used here showed the full range of possible responses to an identical heat perturbation sequence. This diversity of responses could have consequences for soil-borne ecosystem services, highlighting the potential importance of fungal biodiversity in maintaining such services, particularly in the context of climate change.

## Introduction

Climate warming is threatening ecosystems worldwide (IPCC, 2018). Climate change does not only mean increased temperature averages but also increased frequency of extreme events, such as summer heatwaves (Hanson et al., 2006; Bárcenas-Moreno et al., 2009; Frank et al., 2015; IPCC, 2018). Such extremes can profoundly influence individual physiological performance and fitness, phenotypic plasticity, demography and population dynamics, species interactions, and community structure (Vázquez et al., 2015 and references therein), probably even more so than an increase in mean conditions (Thompson et al., 2013).

Performances of soil microbes under an elevated average temperature have been widely investigated (Hortal et al., 2016), but the responses to heat pulse perturbations are understudied (Jentsch et al., 2007; Kreyling and Beier, 2013). Temperature pulse perturbations occurring within a short period of time can be especially damaging, because soil organisms may not be able to adjust their physiological response fast enough (Alley et al., 2003; Fischer and Knutti, 2015). Nevertheless, most temperature-related experimental designs have minimized temperature variability to solely focus on the effects of one average temperature (Lloret et al., 2012; Thompson et al., 2013). Understanding how heat pulse perturbations affect soil microbial performance is an important issue in soil ecology that could lead to a better understanding of aboveground and belowground community functioning.

In particular, the responses to multiple perturbations are far less understood but important, since multiple events may result in diverse response types because the effect size of a single event may depend on the antecedent event, known as ecological memory or carryover effect (Ryo et al., 2019). Considering the growing threat of recurrent heatwaves, it has been recently advocated that experiments aimed at investigating the impact of extreme weather events should consider that today’s extremes will become the normal fluctuations in the future; and experimental designs should exceed the level of severity that we currently observe to provide an insight into an organism’s responses to conditions harsher than those under which they evolved (Bahn et al., 2014; Kayler et al., 2015; Foster et al., 2016). Nevertheless, how soil saprobic fungi respond to recurrent temperature pulse perturbations is largely unknown. This is a large gap, since soil filamentous fungi are sensitive to global change (Rillig et al., 2019) and are important players in many soil processes, including decomposition, respiration and soil aggregation.

The combination of multiple stressors (perturbations) can result in additive effects, detrimental effects (i.e., synergism) or cause a reduction in effects (i.e., antagonism) (Mittler, 2006). Additive effects means that stressors do not interact and therefore the combined effect is simply the sum of each effect. Synergy results from a positive interaction, exceeding the sum of negative effects caused by each single stress event (Côté et al., 2016). Antagonism means that the combined effect is lower than the sum of each (negative) effect, such as observed in the form of stress priming ability (Rillig et al., 2015; Hilker et al., 2016). Priming ability means that a first exposure to a milder stress event induces protection mechanisms, consequently alleviating the effect of a subsequent stronger stress event (Rillig et al., 2015; Andrade-Linares et al., 2016; Hilker et al., 2016). While such different response types are theoretically possible, there is no study testing if such diverse responses to recurrent heat pulses are present in soil microbes co-occurring in the same environment. Additionally, studies on pulse temperature perturbations focus mostly on the community perspective, not providing information on species-level physiological responses (Norris et al., 2002; Allison and Martiny, 2008; Crowther and Bradford, 2013; Bérard et al., 2015; Zhou et al., 2017).

The purpose of the present study was to investigate the diversity of growth responses of soil filamentous fungi to sequential high-temperature pulses exceeding current adverse extreme conditions. We investigated how recurrent temperature pulses affect the performance of individual fungi from a set of 32 soil filamentous fungi that had been isolated from the same soil. We exposed fungi to one or two high-temperature pulse perturbations differing in magnitude [35°C/2 h – mild (M), 45°C/1 h – severe (S), and the sequence of these two perturbations 35°/2 h + 45°C/1 h (MS)] and measured growth responses (colony extension rates). We expected the following: (1) exposure of soil saprobic fungi to recurrent temperature pulses will lead to diverse, isolate-specific responses and (2) the diversity of responses to recurrent pulse temperature disturbance is phylogenetically conserved.

## Materials and Methods

### Fungal Isolates

Isolates of 32 soil fungi were originally cultured from the top 10 cm of soil in a semi-arid grassland in Mallnow Lebus, Brandenburg, Germany (Andrade-Linares et al., 2016). Fungi are referred to by their strain number here; for more detail on these strains see Lehmann et al. (2019) and Supplementary Table S1. We chose this number of isolates to cover major groups (Basidiomycota, Ascomycota, and Mucoromycota), while still having a manageable set of fungi with which to conduct experiments. To obtain material for the experiment, 6.5 mm plugs were taken from the edge of fungal colonies and placed centrally on 9 cm-diameter Petri dishes with potato dextrose agar (PDA) medium. Plates were then incubated at 22°C for 1–5 days, depending on individual colony extension rates, to obtain fresh and actively growing material for inoculation. Then, fungi were re-inoculated on fresh PDA plates and placed in incubators for the experiment.

### Heat Treatment

In the field where the fungi were collected, the topsoil (at approx. 10 cm depth) temperature recorded in the year 2018 (52°52.778′N, 14°29.349′E) (Andrade-Linares et al., 2016) reached 32°C (Andrade-Linares et al., 2016; Dr. Max-Bernhard Ballhausen, personal information, data not shown). We used 35°C/2 h as the mild perturbation (M) pulse temperature, since it is a temperature outside of the range of optimal growth conditions for half of the tested isolates, and it resulted in growth reduction in half of the tested fungi (Andrade-Linares et al., 2016). As the severe perturbation (S), we used 45°C applied for 1 h since the responses to this temperature were severe for most of the isolates in our set (Andrade-Linares et al., 2016).

The full factorial experiment consisted of the following treatments: control (C) 22°C; mild perturbation (M) (35°C/2 h); severe perturbation (S) (45°C/1 h); and sequence of the two perturbations (MS) (35°C/2 h+45°C/1 h).

Temperature pulses were applied uniformly to all 32 soil saprobic fungi. Each treatment consists of three replicates, with incubators used as experimental units. First, samples were incubated for 2–6 days to allow a fungal colony to begin growing from the inoculated plug. Then fungi were exposed to the different pulse temperature perturbations.

### Trait Measurements

The colony diameter of each isolate was measured for each Petri dish in two directions, at right angles to each other. Such measurements were taken four times – the first time before starting heat treatments to determine initial colony size, and then three more times after the treatment to define the response to heat exposure. The frequency of diameter measurements was isolate dependent and taken daily for fast-growing fungi or every 2–4 days for slow-growing individuals.

Thus, in total 1,536 data points were acquired [i.e., 32 isolates × (2 temperatures magnitudes (M,S) + 1 temperature combination (MS) + control (C)) × 3 replicates × 4 time points]. The diameter was measured repeatedly to calculate colony extension rate (mm day^–1^).

### Statistical Analyses

Colony extension rates after heat treatments were used as a response variable to applied temperature pulses. Treatment effects were tested with two-way ANOVA where factors were the applied temperature regimes: mild perturbation (M, yes/no) and stronger perturbation (S, yes/no). Note that the no–no combination indicates control, while the yes–yes combination indicates two perturbations. The significance level α was set to 0.05 with Benjamini–Hochberg correction, to control for experiment-wise type I error rate.

**FIGURE 1:**
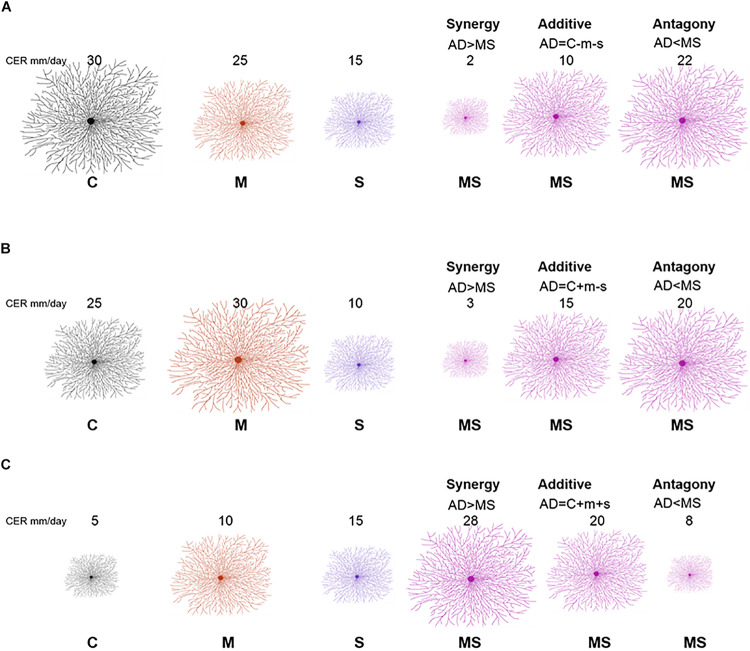
Redrawn from Crain et al. (2008) with adjusted conceptual approach for interpreting interaction types from factorial experiment response data. A full factorial study includes the following treatments: control (C), mild perturbation (M), strong perturbation (S) and both (MS). The three panels illustrate different combinations of individual responses: double negative **(A)**, opposing **(B)**, and double positive **(C)**. The numbers indicate example values of colony extension rate (proportional to the size of the mycelium). Interaction types (synergy, additive, and antagony) depend on MS response in comparison with the sum (AD) of individual responses of single perturbations (*m* = *C* − *M*; s = *C* − *S*).

We used this analysis to classify responses of fungi. Specifically, response classification is based on additive null model expectations, used to identify interactions (antagony, synergy, and additivity) between multiple perturbations (Crain et al., 2008; Côté et al., 2016). The additive null model has been reported to fit responses such as growth (colony extension) of an organism and is consistent with the use of ANOVA for factorial experimental data (Piggott et al., 2015; Côté et al., 2016). Response types were assigned to three groups (see Table 1). The effect direction of the two single perturbation effects in this study could be double negative (both single perturbations reduce the growth rate of the fungal isolate), opposing (one single perturbation increases the growth rate and the other single perturbation decreases the growth rate of the fungal isolate) or double positive (both single perturbations increase the growth rate of the fungal isolate) (Figure 1). Those effect directions are crucial to assign a response to interaction types (Crain et al., 2008).

**TABLE 1:** Interaction types describing the outcome of multiple stressors (perturbations) following Crain et al. (2008) and Côté et al. (2016).

	**Individual treatment response**		
	**Additive**	**Non-additive**	
		**Synergy**	**Antagony**
Condition	No significant interaction term between milder perturbation (M) and stronger perturbation (S)	A significant interaction term between milder perturbation (M) and stronger perturbation (S)	A significant interaction term between milder perturbation (M) and stronger perturbation (S)
Outcome category	The effect of MS is the equivalent of the addition of the single effects of M and S	The effect of MS is stronger than the added effects of M and S	The effect of MS is weaker than the added effects of M and S

*M, milder perturbation; S, stronger perturbation; MS, both perturbations applied.*

In addition, our study can be viewed in the context of stress priming, for which criteria were previously established (Andrade-Linares et al., 2016): (1) negative effect of strong perturbation (S); (2) significant interaction between mild and strong perturbations; and (3) the interaction term has a positive sign (MS > S).

We tested for phylogenetic signal for the measured trait categories using the phylosignal R package. We tested the null hypothesis of absence of signal (i.e., trait values are randomly distributed in the phylogeny) for five phylogenetic signal measures: The Moran’s I index, the Abouheif’s Cmean index, Blomberg’s K and K^∗^ and Pagel’s k.

## Results

**FIGURE 2:**
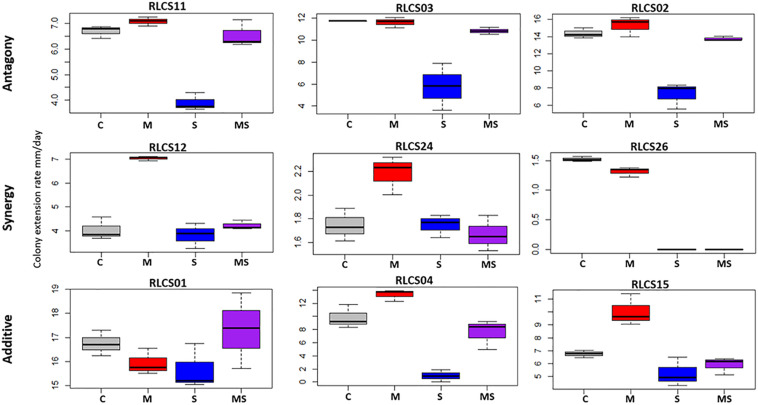
Examples of response categories (based on colony extension rate) to applied recurrent heat pulse perturbations: antagony (shown are 3 of the 13 isolates so categorized), synergy (all 3 isolates in this category are shown), additive (3 of the 16 isolates so categorized are depicted). For a full figure containing data for all 32 isolates see Supplementary Figure S1.

**FIGURE 3:**
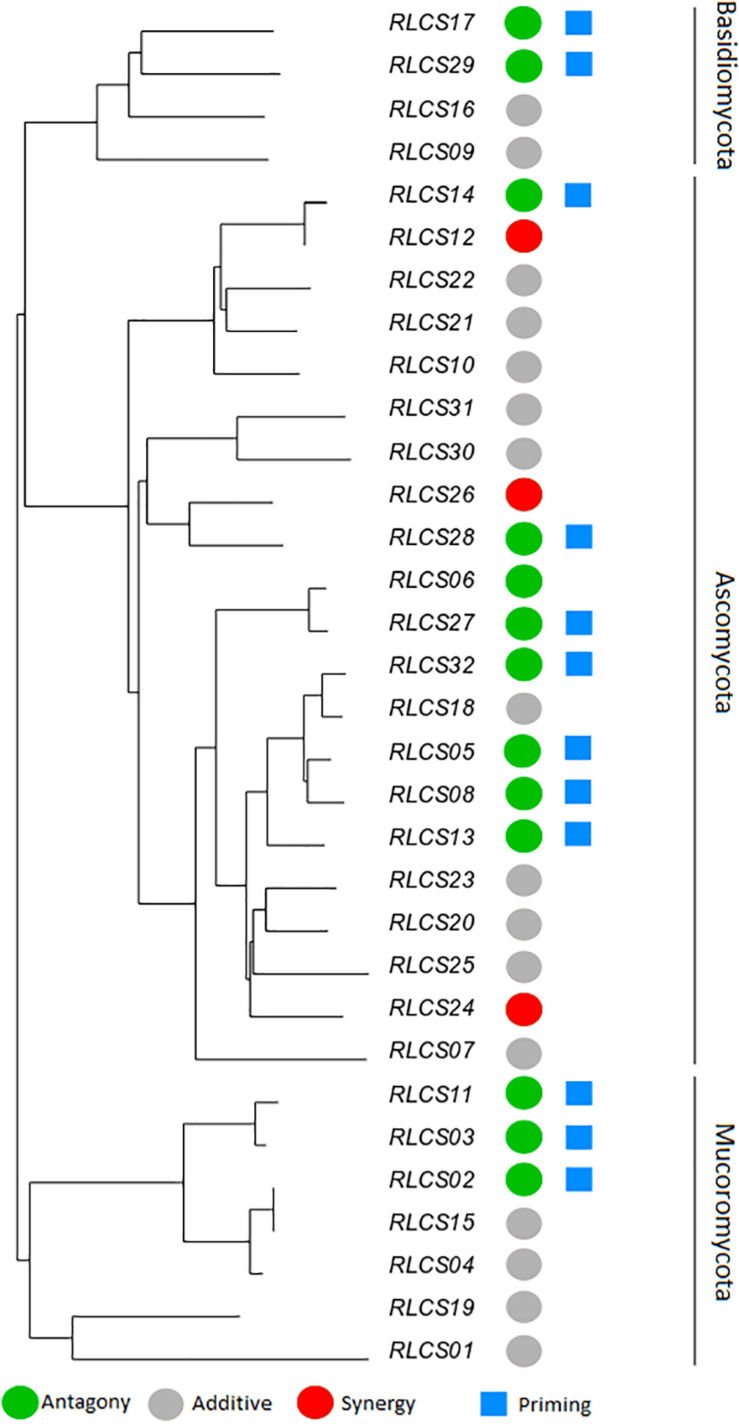
Phylogenetic relationship of a set of soil fungal isolates originating from the same field site with the representation of diverse responses of fungal colony extension rate to recurrent heat pulse perturbations (2 h mild perturbation 35°C + 1 h strong perturbation 45°C). The colored circles represent response types: antagony (green) (13 isolates); synergy (red) (3 isolates); additive (gray) (16 isolates). Blue squares mark isolates (12) that showed priming ability [based on criteria of Andrade-Linares et al. (2016)]. The remaining isolates (19) did not meet the criteria of priming ability. The neighbor-joining tree was based on ITS and LSU regions; detailed information on isolates is in Supplementary Table S1.

Fungal isolates showed a range of responses to the applied sequences of temperature perturbation (Figures 2, 3). There was a significant interaction term for the two perturbations (M:S) in 16 tested isolates, and these were further categorized as antagonistic (13 isolates) or synergistic (3 isolates; Table 2). Isolates that did not meet the criterion of a significant interaction term (M:S) were assigned to the category ‘additive’ (16 isolates) (Tables 1, 2).

**TABLE 2:** Analysis of variance results of effects of mild (M, yes/no), strong (S, yes/no), and both heat stress events (MS).

**Isolate**	**Source**	**df**	**Sum Sq**	***F***	***p*-value (Benjamini–Hochberg corrected)**
RLCS09	Mild	1	0.00	0.00	0.98
	Strong	1	0.00	0.01	0.97
	Mild:Strong	1	0.01	0.05	0.85
	Residuals	8	1.32		
RLCS16	Mild	1	0.20	0.64	0.57
	Strong	1	0.60	1.89	0.24
	Mild:Strong	1	0.02	0.06	0.85
	Residuals	8	2.54		
RLCS17	Mild	1	0.19	1.94	0.38
	Strong	1	1.84	19.16	**0.01**
	Mild:Strong	1	1.45	15.09	**0.01**
	Residuals	8	0.77		
RLCS29	Mild	1	0.00	0.01	0.96
	Strong	1	1.25	61.86	**0.00**
	Mild:Strong	1	0.51	25.24	**0.00**
	Residuals	8	0.16		
RLCS10	Mild	1	1.36	10.76	**0.03**
	Strong	1	1.16	9.22	**0.02**
	Mild:Strong	1	0.82	6.49	0.06
	Residuals	8	1.01		
RLCS14	Mild	1	1.40	26.14	**0.00**
	Strong	1	0.15	2.72	0.18
	Mild:Strong	1	0.79	14.74	**0.01**
	Residuals	8	0.43		
RLCS12	Mild	1	8.66	63.93	**0.00**
	Strong	1	6.84	50.45	**0.00**
	Mild:Strong	1	5.02	37.04	**0.00**
	Residuals	8	1.08		
RLCS22	Mild	1	0.01	0.23	0.73
	Strong	1	0.23	6.91	**0.04**
	Mild:Strong	1	0.07	2.05	0.24
	Residuals	8	0.27		
RLCS21	Mild	1	0.26	1.64	0.40
	Strong	1	7.05	44.20	**0.00**
	Mild:Strong	1	0.47	2.93	0.18
	Residuals	8	1.28		
RLCS31	Mild	1	0.00	0.40	0.65
	Strong	1	0.84	119.36	**0.00**
	Mild:Strong	1	0.03	3.95	0.12
	Residuals	8	0.06		
RLCS30	Mild	1	0.02	1.42	0.43
	Strong	1	1.72	102.66	**0.00**
	Mild:Strong	1	0.01	0.59	0.55
	Residuals	8	0.13		
RLCS26	Mild	1	0.03	15.26	**0.01**
	Strong	1	6.04	2933.90	**0.00**
	Mild:Strong	1	0.03	15.26	**0.01**
	Residuals	8	0.02		
RLCS28	Mild	1	0.01	1.04	0.51
	Strong	1	0.82	123.11	**0.00**
	Mild:Strong	1	0.47	69.89	**0.00**
	Residuals	8	0.05		
RLCS07	Mild	1	3.20	10.42	**0.03**
	Strong	1	5.36	17.43	**0.01**
	Mild:Strong	1	0.04	0.12	0.84
	Residuals	8	2.46		
RLCS06	Mild	1	2.02	7.87	**0.05**
	Strong	1	4.91	19.11	**0.01**
	Mild:Strong	1	8.50	33.07	**0.01**
	Residuals	8	2.06		
RLCS27	Mild	1	0.06	0.99	0.51
	Strong	1	0.05	0.77	0.45
	Mild:Strong	1	3.43	55.08	**0.00**
	Residuals	8	0.50		
RLCS13	Mild	1	0.00	0.01	0.96
	Strong	1	0.55	11.29	**0.02**
	Mild:Strong	1	1.20	24.41	**0.00**
	Residuals	8	0.39		
RLCS32	Mild	1	4.35	26.68	**0.00**
	Strong	1	7.73	47.34	**0.00**
	Mild:Strong	1	23.84	146.09	**0.00**
	Residuals	8	1.31		
RLCS18	Mild	1	2.02	4.92	0.11
	Strong	1	0.00	0.00	1.00
	Mild:Strong	1	1.02	2.49	0.20
	Residuals	8	3.29		
RLCS05	Mild	1	1.26	25.75	**0.00**
	Strong	1	0.30	6.06	**0.05**
	Mild:Strong	1	15.64	320.18	**0.00**
	Residuals	8	0.39		
RLCS08	Mild	1	3.75	33.25	**0.00**
	Strong	1	1.68	14.92	**0.01**
	Mild:Strong	1	2.78	24.68	**0.00**
	Residuals	8	0.90		
RLCS24	Mild	1	0.10	5.25	0.11
	Strong	1	0.20	10.26	**0.02**
	Mild:Strong	1	0.20	10.59	**0.02**
	Residuals	8	0.15		
RLCS23	Mild	1	0.65	0.84	0.52
	Strong	1	3.97	5.13	0.07
	Mild:Strong	1	0.04	0.06	0.85
	Residuals	8	6.18		
RLCS20	Mild	1	0.09	1.84	0.38
	Strong	1	0.05	1.02	0.39
	Mild:Strong	1	0.29	6.31	0.06
	Residuals	8	0.37		
RLCS25	Mild	1	0.41	0.89	0.52
	Strong	1	1.13	2.43	0.19
	Mild:Strong	1	0.69	1.49	0.32
	Residuals	8	3.70		
RLCS11	Mild	1	6.85	55.56	**0.00**
	Strong	1	8.29	67.20	**0.00**
	Mild:Strong	1	3.75	30.43	**0.00**
	Residuals	8	0.99		
RLCS03	Mild	1	18.31	15.27	**0.01**
	Strong	1	34.08	28.43	**0.00**
	Mild:Strong	1	20.34	16.96	**0.01**
	Residuals	8	9.59		
RLCS04	Mild	1	76.09	30.59	**0.00**
	Strong	1	76.09	30.59	**0.00**
	Mild:Strong	1	7.16	2.88	0.18
	Residuals	8	19.90		
RLCS02	Mild	1	40.87	40.32	**0.00**
	Strong	1	56.10	55.34	**0.00**
	Mild:Strong	1	23.09	22.78	**0.00**
	Residuals	8	8.11		
RLCS15	Mild	1	11.36	13.86	**0.02**
	Strong	1	23.69	28.90	**0.00**
	Mild:Strong	1	5.09	6.21	0.06
	Residuals	8	6.56		
RLCS01	Mild	1	0.52	0.53	0.60
	Strong	1	0.07	0.07	0.85
	Mild:Strong	1	4.56	4.62	0.10
	Residuals	8	7.90		
RLCS19	Mild	1	0.00	0.02	0.96
	Strong	1	2.30	9.93	**0.02**
	Mild:Strong	1	0.01	0.03	0.87
	Residuals	8	1.62		

*The full results table is available in Supplementary Table S2. Bold indicates p < 0.05.*

The observed antagonistic response is largely congruent (with small differences in categorization occurring, since two different statistical approaches are used – one isolate was classified differently) with what defines the priming ability of an organism (Andrade-Linares et al., 2016). We found priming responses in 12 out of the 32 isolates – 3 Mucoromycotina, 2 Basidiomycota, and 7 Ascomycota. That is, for these 12 isolates, growth was enhanced when fungi were exposed to a mild temperature pulse (M) before the severe temperature (MS), compared to when they were only experiencing the severe pulse (S). For the remaining isolates exposure to experimental conditions of sequential perturbation (MS) did not lead to increased performance. Priming ability, antagonist response, additive response or synergistic response did not show a phylogenetic signal for any of the phylogenetic indices (Table 3).

**TABLE 3:** Phylogenetic signal test for priming ability (PRI), antagonist response (ANT), additive response (ADD), and synergistic response (SYN).

	**Cmean**	**I**	**K**	**K.star**	**Lambda**
**Statistics**					
PRI	0.0439	–0.0906	0.0420	0.0478	5.385e-05
ANT	0.1105	–0.0604	0.0458	0.0514	6.37e-05
ADD	0.1413	–0.0404	0.0522	0.0573	7.97e-05
SYN	–0.0959	–0.0481	0.1514	0.1733	7.97e-05
***P*-values**					
PRI	0.274	0.783	0.901	0.883	1
ANT	0.129	0.603	0.726	0.716	1
ADD	0.082	0.538	0.521	0.513	1
SYN	0.665	0.679	0.715	0.723	1

## Discussion

Here we show that exposure of soil saprobic fungi isolates to the same sequence of pulse temperature perturbations results in a range of response types that include additive effect, antagony and synergy. The applied temperature pulse regime was exceeding the temperatures that are currently typically observed at the site from which isolates were obtained. The biggest proportion of isolates (16/32) responded in an additive manner to the perturbations. The synergistic response (two perturbations leading to decreased performance) was observed for 3/32 isolates, meaning that such a sequence of perturbations could limit rates of processes (e.g., decomposition) these species carry out. On the other hand, the same temperature pulse regime resulted in an antagonistic response (two perturbations leading to an increased performance of an organism) for over 40% of isolates.

The observed antagonistic response of 12 isolates is congruent with priming ability of isolates. Priming ability of eight filamentous fungi isolates exposed to a rather different temperature sequence (5 h/35°C, then 40°C/10 h) has been shown previously (Andrade-Linares et al., 2016). The fact that a large proportion of our fungi showed this improved response to sequential stresses, even under different temperature regimes, may indicate that this priming ability is not an exceptional response, but rather a well-established phenomenon that can help fungi deal with adverse effects.

In addition, the observed exacerbation of growth inhibition of three treated isolates (RLCS06, RLCS12, and RLCS24) due to sequential heat exposure has not been observed before. For these fungi, the first heat stress event clearly did not help them in dealing with the second, more severe heat pulse. These would therefore be interesting targets to study further in the context of climate change and heat extremes.

At the species level, physiological stress regimes are known to set biogeographic limits and determine microhabitat preferences. Organismal responses to extreme heat events include redirecting resources from growth to survival that may include transition to a dormancy state or sporulation. These variations in heat responses are species specific and may be caused by differences in cellular HSP (heat shock proteins) production, altered membrane composition and carbohydrate flux (Morano et al., 2012; Bérard et al., 2015). The difference in response to sequential temperature pulse perturbation in isolates originating from one fungal assembly may indicate differences in sensitivity and diverse stress tolerances.

Our results show that recurrent environmental perturbations such as extreme temperature events influence a group of soil filamentous fungi originating from the same site in various ways. Thus, the patterns of responses that they exhibit to the sequence of thermal pulses might be one of the factors that contribute to shaping soil fungal community composition. Such differences in aspects of the ‘thermal niche’ may contribute to coexistence of fungi in the community, much like differences among species in other abiotic factors or resource utilization patterns.

## Conclusion

We focused on growth responses of multiple isolates of soil filamentous fungi, originating from the same grassland to a sequence of thermal pulses. These fungal isolates, isolated from the same soil, revealed the full range of possible responses to an identical heat perturbation sequence. This diversity of responses could have consequences for soil-borne ecosystem processes, highlighting the potential importance of fungal biodiversity in maintaining such services, particularly in the context of climate change.

## Author’s Note

An earlier version of this manuscript has been released as a Pre-Print at bioRxiv (Szymczak et al., 2019).

## Data Availability Statement

The raw data supporting the conclusions of this article will be made available by the authors, without undue reservation, to any qualified researcher.

## Author Contributions

AS and MCR designed the research and wrote the manuscript. AS performed the research. AS, JR, and MR performed the data analyses. All authors contributed to the final version of the manuscript.

## Conflict of Interest

The authors declare that the research was conducted in the absence of any commercial or financial relationships that could be construed as a potential conflict of interest.

## References

[B1] Alley R. B. Marotzke J. Nordhaus W. D. Overpeck J. T. Peteet D. M. Pielke R. A. (2003). Abrupt climate change. *Science* 299 2005–2010. 10.1126/science.1081056 12663908

[B2] Allison S. D. Martiny J. B. H. (2008). Resistance, resilience, and redundancy in microbial communities. *Proc. Natl. Acad. Sci. U.S.A.* 105(Suppl. 1), 11512–11519. 10.1073/pnas.0801925105 18695234 PMC2556421

[B3] Andrade-Linares D. R. Veresoglou S. D. Rillig M. C. (2016). Temperature priming and memory in soil filamentous fungi. *Fungal Ecol.* 21 10–15. 10.1016/j.funeco.2016.02.002

[B4] Bahn M. Reichstein M. Dukes J. S. Smith M. D. Mcdowell N. G. (2014). Climate-biosphere interactions in a more extreme world. *New Phytol.* 202 356–359. 10.1111/nph.12662 24383455

[B5] Bárcenas-Moreno G. Brandón M. G. Rousk J. Bååth E. (2009). Adaptation of soil microbial communities to temperature: comparison of fungi and bacteria in a laboratory experiment. *Glob. Change Biol.* 15 2950–2957. 10.1111/j.1365-2486.2009.01882.x

[B6] Bérard A. Ben Sassi M. Kaisermann A. Renault P. (2015). Soil microbial community responses to heat wave components: drought and high temperature. *Clim. Res.* 66 243–264. 10.3354/cr01343

[B7] Côté I. M. Darling E. S. Brown C. J. (2016). Interactions among ecosystem stressors and their importance in conservation. *Proc. R. Soc. B Biol. Sci.* 283:20152592. 10.1098/rspb.2015.2592 26865306 PMC4760168

[B8] Crain C. A. Kroeker K. Halpern B. S. (2008). Interactive and cumulative effects of multiple human stressors in marine systems. *Ecol. Lett.* 11 1304–1315. 10.1111/j.1461-0248.2008.01253 19046359

[B9] Crowther T. W. Bradford M. A. (2013). Thermal acclimation in widespread heterotrophic soil microbes. *Ecol. Lett.* 16 469–477. 10.1111/ele.12069 23331708

[B10] Fischer E. M. Knutti R. (2015). Anthropogenic contribution to global occurrence of heavy-precipitation and high-temperature extremes. *Nat. Clim. Change* 5 560–564. 10.1038/nclimate2617

[B11] Foster C. N. Sato C. F. Lindenmayer D. B. Barton P. S. (2016). Integrating theory into disturbance interaction experiments to better inform ecosystem management. *Glob. Change Biol.* 22 1325–1335. 10.1111/gcb.13155 26554638

[B12] Frank D. Reichstein M. Bahn M. Thonicke K. Frank D. Mahecha M. D. (2015). Effects of climate extremes on the terrestrial carbon cycle: concepts, processes and potential future impacts. *Glob. Change Biol.* 21 2861–2880. 10.1111/gcb.12916 25752680 PMC4676934

[B13] Hanson C. E. Palutikof J. P. Dlugolecki A. Giannakopoulos C. (2006). Bridging the gap between science and the stakeholder: the case of climate change research. *Clim. Res.* 31 121–133. 10.3354/cr031121

[B14] Hilker M. Schwachtje J. Baier M. Balazadeh S. Bäurle I. Geiselhardt S. (2016). Priming and memory of stress responses in organisms lacking a nervous system. *Biol. Rev.* 49 1118–1133. 10.1111/brv.12215 26289992

[B15] Hortal S. Powell J. R. Plett J. M. Simonin A. Anderson I. C. (2016). Intraspecific competition between ectomycorrhizal Pisolithus microcarpus isolates impacts plant and fungal performance under elevated CO_2_ and temperature. *FEMS Microbiol. Ecol.* 92:fiw113. 10.1093/femsec/fiw113 27222224

[B16] IPCC (2018). *Global Warming of 1.5°C. An IPCC Special Report on the Impacts of Global Warming of 1.5°C Above Pre-Industrial Levels and Related Global Greenhouse Gas Emission Pathways, in the Context of Strengthening the Global Response to the Threat of Climate Change, Sustainable Development, and Efforts to Eradicate Poverty*, eds Masson-Delmotte V. Zhai P. Pörtner H. O. Roberts D. Skea J. Shukla P. R. (Geneva: IPCC).

[B17] Jentsch A. Kreyling J. Beierkuhnlein C. (2007). A new generation of events, not trends experiments. *Front. Ecol. Environ.* 5 365–374. 10.1890/1540-929520075[365:ANGOCE]2.0.CO;2

[B18] Kayler Z. E. De Boeck H. J. Fatichi S. Grünzweig J. M. Merbold L. Beier C. (2015). Experiments to confront the environmental extremes of climate change. *Front. Ecol. Environ.* 13 219–225. 10.1890/140174

[B19] Kreyling J. Beier C. (2013). Complexity in climate change manipulation experiments. *Bioscience* 63 763–767. 10.1525/bio.2013.63.9.12

[B20] Lehmann A. Zheng W. Soutschek K. Roy J. Yurkov A. Rillig M. C. (2019). Tradeoffs in hyphal traits determine mycelium architecture in saprobic fungi. *Sci. Rep.* 9:14152. 10.1038/s41598-019-50565-7 31578362 PMC6775140

[B21] Lloret F. Escudero A. Iriondo J. M. Martinez-Vilalta J. Valladarez F. (2012). Extreme climatic events and vegetation: the role of stabilizing processes. *Glob. Change Biol.* 18 797–805. 10.1111/j.1365-2486.2011.02624.x

[B22] Mittler R. (2006). Abiotic stress, the field environment and stress combination. *Trends Plant Sci.* 11 15–19. 10.1016/j.tplants.2005.11.002 16359910

[B23] Morano K. A. Grant C. M. Moye-Rowley W. S. (2012). The response to heat shock and oxidative stress in saccharomyces cerevisiae. *Genetics* 190 1157–1195. 10.1534/genetics.111.128033 22209905 PMC3316637

[B24] Norris T. B. Wraith J. M. Castenholz R. W. McDermott T. R. (2002). Soil microbial community structure across a thermal gradient following a geothermal heating event. *Appl. Environ. Microbiol.* 68 6300–6309. 10.1128/AEM.68.12.6300-6309.2002 12450855 PMC134386

[B25] Piggott J. J. Townsend C. R. Matthaei C. D. (2015). Reconceptualizing synergism and antagonism among multiple stressors. *Ecol. Evol.* 5 1538–1547. 10.1002/ece3.1465 25897392 PMC4395182

[B26] Rillig M. C. Rolff J. Tietjen B. Wehner J. Andrade-Linares D. R. (2015). Community priming—effects of sequential stressors on microbial assemblages. *FEMS Microbiol. Ecol.* 91:fiv040. 10.1093/femsec/fiv040 25873462

[B27] Rillig M. C. Ryo M. Lehmann A. Aguilar-Trigueros C. A. Buchert S. Wulf A. (2019). The role of multiple global change factors in driving soil functions and microbial biodiversity. *Science* 366 886–890. 10.1126/science.aay2832 31727838 PMC6941939

[B28] Ryo M. Aguilar-Trigueros C. A. Pinek L. Muller L. A. H. Rillig M. C. (2019). Basic principles of temporal dynamics. *Trends Ecol. Evol.* 34 723–733. 10.1016/j.tree.2019.03.007 31010706

[B29] Szymczak A. Ryo M. Rillig M. C. (2019). Diversity of responses of soil saprobic fungi to recurring heat events. *BioRxiv* [Preprint]. 10.1101/733923 PMC7316893 32636822

[B30] Thompson R. M. Beardall J. Beringer J. Grace M. Sardina P. (2013). Means and extremes: building variability into community-level climate change experiments. *Ecol. Lett.* 16 799–806. 10.1111/ele.12095 23438320

[B31] Vázquez D. P. Gianoli E. Morris W. F. Bozinovic F. (2015). Ecological and evolutionary impacts of changing climatic variability. *Biol. Rev.* 92 22–43. 10.1111/brv.12216 26290132

[B32] Zhou W. P. Shen W. J. Li Y. E. Hui D. F. (2017). Interactive effects of temperature and moisture on composition of the soil microbial community. *Eur. J. Soil Sci.* 68 909–918. 10.1111/ejss.12488

