# Supplementary material for: Diversity of Growth Responses of Soil Saprobic Fungi to Recurring Heat Events
Source: Front Microbiol. 2020 Jun 19;11:1326. doi: 10.3389/fmicb.2020.01326 (PMC7316893; doi:10.3389/fmicb.2020.01326)

**Supplementary material**

Table S1. Taxonomic identification of the soil filamentous fungi used in this study, and accession numbers. The order of species in the table is by phylogeny. The neighbor-joining tree was based on the ITS (intergenic transcribed spacer) and a part of the large rRNA subunit (LSU). Phylogenetic annotations were based on bootstrap analysis, and assumed valid when supported in 80% of the bootstraps.

| **Strain ID** | **Genus species** | **Order** | **Phylum** | **NCBI Accession number** | **DSMZ** | **Partial 18s-, full ITS-, partial LSU sequence accession number** |
| --- | --- | --- | --- | --- | --- | --- |
| RLCS09 | *Trametes versicolor* | Polyporales | Basidiomycota | KT582071 | DSM 100406 | MT453293 |
| RLCS16 | *Pleurotus sapidus* | Agaricales | Basidiomycota | KT582080 | DSM 100408 | MT453295 |
| RLCS17 | *Clitopilus sp.* | Agaricales | Basidiomycota | KT582089 | DSM 100324 | MT453280 |
| RLCS29 | *Macrolepiota excoriata* | Agaricales | Basidiomycota | KT582069 | DSM 100288 | MT453273 |
| RLCS10 | *Alternaria alternata* | Pleosporales | Ascomycota | KT582078 | DSM 100286 | MT453271 |
| RLCS14 | *Didymellaceae*  strain 2 | Pleosporales | Ascomycota | KT582077 | DSM 100404 | MT453301 |
| RLCS12 | *Didymellaceae*  strain 1 | Pleosporales | Ascomycota | KT582079 | DSM 100405 | MT453292 |
| RLCS22 | *Paraphoma chrysanthemicola* | Pleosporales | Ascomycota | KT582091 | DSM 100401 | MT453289 |
| RLCS21 | *Pyrenochaetopsis leptospora* | Pleosporales | Ascomycota | KT582065 | DSM 100327 | MT453283 |
| RLCS31 | *Cyphellophora sp.* | Chaetothyriales | Ascomycota | KT582074 | DSM 100328 | MT453284 |
| RLCS30 | *Exophiala equina* | Chaetothyriales | Ascomycota | KT582075 | DSM 100291 | MT453276 |
| RLCS26 | *Tetracladium marchalianum* | Helotiales | Ascomycota | KT582084 | DSM 100330 | MT453286 |
| RLCS28 | *Tricladium sp.* | Helotiales | Ascomycota | KT582085 | DSM 100323 | MT453279 |
| RLCS07 | *Amphisphaeriaceae*  strain 1 | Xylariales | Ascomycota | KT582088 | DSM 100284 | MT453269 |
| RLCS06 | *Chaetomium angustispirale* | Sordariales | Ascomycota | KT582096 | DSM 100400 | MT453288 |
| RLCS27 | *Thielavia inaequalis* | Sordariales | Ascomycota | KT582086 | DSM 100326 | MT453282 |
| RLCS13 | *Fusarium solani* | Hypocreales | Ascomycota | KT582073 | DSM 100290 | MT453275 |
| RLCS32 | *Fusarium oxysporum* | Hypocreales | Ascomycota | KT582095 | DSM 100409 | MT453296 |
| RLCS18 | *Gibberella sp.* | Hypocreales | Ascomycota | KT582068 | DSM 100287 | MT453272 |
| RLCS05 | *Fusarium sp.* | Hypocreales | Ascomycota | KT582097 | DSM 100403 | MT453291 |
| RLCS08 | *Gibberella tricincta* | Hypocreales | Ascomycota | KT582087 | DSM 100325 | MT453281 |
| RLCS24 | *Metarhizium marquandii* | Hypocreales | Ascomycota | KT582066 | DSM 100410 | MT453297 |
| RLCS23 | *Stachybotryaceae*  strain 1 | Hypocreales | Ascomycota | KT582090 | DSM 101519 | MT453299 |
| RLCS20 | *Purpureocillium lilacinum* | Hypocreales | Ascomycota | KT582081 | DSM 100329 | MT453285 |
| RLCS25 | *Hydropisphaera* sp. | Hypocreales | Ascomycota | KT582083 | DSM 100292 | MT453277 |
| RLCS11 | *Mortierella alpina*  strain 2 | Mortierellales | Mucoromycota | KT582070 | DSM 100289 | MT453274 |
| RLCS03 | *Mortierella alpina*  strain 1 | Mortierellales | Mucoromycota | KT582067 | DSM 100285 | MT453270 |
| RLCS04 | *Mortierella exigua* | Mortierellales | Mucoromycota | KT582094 | DSM 100322 | MT453300 |
| RLCS02 | *Mortierella elongata*  strain 1 | Mortierellales | Mucoromycota | KT582072 | DSM 100407 | MT453294 |
| RLCS15 | *Mortierella elongata*  strain 2 | Mortierellales | Mucoromycota | KT582092 | DSM 100402 | MT453290 |
| RLCS01 | *Mucor fragilis* | Mucorales | Mucoromycota | KT582076 | DSM 100293 | MT453278 |
| RLCS19 | *Umbelopsis isabellina* | Umbelopsidales | Mucoromycota | KT582093 | DSM 100331 | MT453287 |

Table S2. Analysis of variance (full result table) - effects of mild (M, yes/no), strong (S, yes/no) and both heat stress events (MS).

| **Isolate** | **Source** | **df** | **Sum Sq** | **Mean Sq** | **F** | **p-value** | **p-value** (Benjamini- Hochberg corrected) |
| --- | --- | --- | --- | --- | --- | --- | --- |
| RLCS09 | Mild | 1 | 0.0001 | 0.0001 | 0.0010 | 0.9800 | 0.9804 |
|  | Strong | 1 | 0.0010 | 0.0010 | 0.0060 | 0.9410 | 0.9715 |
|  | Mild:Strong | 1 | 0.0086 | 0.0086 | 0.0520 | 0.8250 | 0.8515 |
|  | Residuals | 8 | 1.3180 | 0.1648 |  |  |  |
| RLCS16 | Mild | 1 | 0.2032 | 0.2032 | 0.6400 | 0.4470 | 0.5719 |
|  | Strong | 1 | 0.6006 | 0.6006 | 1.8910 | 0.2060 | 0.2445 |
|  | Mild:Strong | 1 | 0.0196 | 0.0196 | 0.0620 | 0.8100 | 0.8515 |
|  | Residuals | 8 | 2.5402 | 0.3175 |  |  |  |
| RLCS17 | Mild | 1 | 0.1859 | 0.1859 | 1.9410 | 0.2011 | 0.3762 |
|  | Strong | 1 | 1.8360 | 1.8360 | 19.1610 | 0.0024 | 0.0051 |
|  | Mild:Strong | 1 | 1.4457 | 1.4457 | 15.0890 | 0.0047 | 0.0106 |
|  | Residuals | 8 | 0.7665 | 0.0958 |  |  |  |
| RLCS29 | Mild | 1 | 0.0002 | 0.0002 | 0.0090 | 0.9284 | 0.9583 |
|  | Strong | 1 | 1.2452 | 1.2452 | 61.8610 | 0.0000 | 0.0002 |
|  | Mild:Strong | 1 | 0.5081 | 0.5081 | 25.2420 | 0.0010 | 0.0036 |
|  | Residuals | 8 | 0.1610 | 0.0201 |  |  |  |
| RLCS10 | Mild | 1 | 1.3588 | 1.3588 | 10.755 | 0.0112 | 0.0298 |
|  | Strong | 1 | 1.1644 | 1.1644 | 9.216 | 0.0162 | 0.0246 |
|  | Mild:Strong | 1 | 0.8195 | 0.8195 | 6.486 | 0.0343 | 0.0630 |
|  | Residuals | 8 | 1.0107 | 0.1263 |  |  |  |
| RLCS14 | Mild | 1 | 1.3975 | 1.3975 | 26.143 | 0.0009 | 0.0038 |
|  | Strong | 1 | 0.1453 | 0.1453 | 2.718 | 0.1379 | 0.1764 |
|  | Mild:Strong | 1 | 0.7877 | 0.7877 | 14.737 | 0.0050 | 0.0106 |
|  | Residuals | 8 | 0.4276 | 0.0535 |  |  |  |
| RLCS12 | Mild | 1 | 8.662 | 8.662 | 63.93 | 0.0000 | 0.0012 |
|  | Strong | 1 | 6.836 | 6.836 | 50.45 | 0.0001 | 0.0004 |
|  | Mild:Strong | 1 | 5.019 | 5.019 | 37.04 | 0.0003 | 0.0019 |
|  | Residuals | 8 | 1.084 | 0.135 |  |  |  |
| RLCS22 | Mild | 1 | 0.00792 | 0.00792 | 0.234 | 0.6413 | 0.7329 |
|  | Strong | 1 | 0.23339 | 0.23339 | 6.909 | 0.0302 | 0.0440 |
|  | Mild:Strong | 1 | 0.06915 | 0.06915 | 2.047 | 0.1904 | 0.2437 |
|  | Residuals | 8 | 0.27023 | 0.03378 |  |  |  |
| RLCS21 | Mild | 1 | 0.262 | 0.262 | 1.640 | 0.2362 | 0.3978 |
|  | Strong | 1 | 7.050 | 7.050 | 44.196 | 0.0002 | 0.0005 |
|  | Mild:Strong | 1 | 0.468 | 0.468 | 2.931 | 0.1253 | 0.1783 |
|  | Residuals | 8 | 1.276 | 0.160 |  |  |  |
| RLCS31 | Mild | 1 | 0.0028 | 0.0028 | 0.396 | 0.5470 | 0.6479 |
|  | Strong | 1 | 0.8361 | 0.8361 | 119.363 | 0.0000 | 0.0000 |
|  | Mild:Strong | 1 | 0.0277 | 0.0277 | 3.952 | 0.0820 | 0.1250 |
|  | Residuals | 8 | 0.0560 | 0.0070 |  |  |  |
| RLCS30 | Mild | 1 | 0.0237 | 0.0237 | 1.415 | 0.2680 | 0.4295 |
|  | Strong | 1 | 1.7176 | 1.7176 | 102.664 | 0.0000 | 0.0001 |
|  | Mild:Strong | 1 | 0.0098 | 0.0098 | 0.586 | 0.4660 | 0.5521 |
|  | Residuals | 8 | 0.1338 | 0.0167 |  |  |  |
| RLCS26 | Mild | 1 | 0.031 | 0.031 | 15.26 | 0.0045 | 0.0144 |
|  | Strong | 1 | 6.041 | 6.041 | 2933.9 | 0.0000 | 0.0000 |
|  | Mild:Strong | 1 | 0.031 | 0.031 | 15.26 | 0.0045 | 0.0106 |
|  | Residuals | 8 | 0.016 | 0.002 |  |  |  |
| RLCS28 | Mild | 1 | 0.0069 | 0.0069 | 1.041 | 0.3380 | 0.5079 |
|  | Strong | 1 | 0.8194 | 0.8194 | 123.113 | 0.0000 | 0.0000 |
|  | Mild:Strong | 1 | 0.4651 | 0.4651 | 69.885 | 0.0000 | 0.0003 |
|  | Residuals | 8 | 0.0532 | 0.0067 |  |  |  |
| RLCS07 | Mild | 1 | 3.202 | 3.202 | 10.420 | 0.0121 | 0.0298 |
|  | Strong | 1 | 5.357 | 5.357 | 17.434 | 0.0031 | 0.0062 |
|  | Mild:Strong | 1 | 0.037 | 0.037 | 0.121 | 0.7367 | 0.8419 |
|  | Residuals | 8 | 2.458 | 0.307 |  |  |  |
| RLCS06 | Mild | 1 | 2.021 | 2.021 | 7.868 | 0.0230 | 0.0526 |
|  | Strong | 1 | 4.909 | 4.909 | 19.109 | 0.0024 | 0.0051 |
|  | Mild:Strong | 1 | 8.495 | 8.495 | 33.068 | 0.0004 | 0.0023 |
|  | Residuals | 8 | 2.055 | 0.257 |  |  |  |
| RLCS27 | Mild | 1 | 0.062 | 0.062 | 0.989 | 0.3490 | 0.5079 |
|  | Strong | 1 | 0.048 | 0.048 | 0.772 | 0.4050 | 0.4471 |
|  | Mild:Strong | 1 | 3.431 | 3.431 | 55.078 | 0.0001 | 0.0006 |
|  | Residuals | 8 | 0.498 | 0.062 |  |  |  |
| RLCS13 | Mild | 1 | 0.0005 | 0.0005 | 0.011 | 0.9194 | 0.9583 |
|  | Strong | 1 | 0.5548 | 0.5548 | 11.288 | 0.0099 | 0.0177 |
|  | Mild:Strong | 1 | 1.1999 | 1.1999 | 24.413 | 0.0011 | 0.0036 |
|  | Residuals | 8 | 0.3932 | 0.0491 |  |  |  |
| RLCS32 | Mild | 1 | 4.354 | 4.354 | 26.68 | 0.0009 | 0.0038 |
|  | Strong | 1 | 7.726 | 7.726 | 47.34 | 0.0001 | 0.0004 |
|  | Mild:Strong | 1 | 23.841 | 23.841 | 146.09 | 0.0000 | 0.0000 |
|  | Residuals | 8 | 1.306 | 0.163 |  |  |  |
| RLCS18 | Mild | 1 | 2.020 | 2.0197 | 4.918 | 0.0574 | 0.1148 |
|  | Strong | 1 | 0.000 | 0.0000 | 0.000 | 0.9981 | 0.9981 |
|  | Mild:Strong | 1 | 1.024 | 1.0240 | 2.493 | 0.1530 | 0.2040 |
|  | Residuals | 8 | 3.286 | 0.4107 |  |  |  |
| RLCS05 | Mild | 1 | 1.258 | 1.258 | 25.752 | 0.0010 | 0.0038 |
|  | Strong | 1 | 0.296 | 0.296 | 6.063 | 0.0392 | 0.0545 |
|  | Mild:Strong | 1 | 15.640 | 15.640 | 320.178 | 0.0000 | 0.0000 |
|  | Residuals | 8 | 0.391 | 0.049 |  |  |  |
| RLCS08 | Mild | 1 | 3.749 | 3.749 | 33.25 | 0.0004 | 0.0034 |
|  | Strong | 1 | 1.682 | 1.682 | 14.92 | 0.0048 | 0.0090 |
|  | Mild:Strong | 1 | 2.783 | 2.783 | 24.68 | 0.0011 | 0.0036 |
|  | Residuals | 8 | 0.902 | 0.113 |  |  |  |
| RLCS24 | Mild | 1 | 0.09973 | 0.0997 | 5.246 | 0.0512 | 0.1093 |
|  | Strong | 1 | 0.19504 | 0.1950 | 10.259 | 0.0126 | 0.0211 |
|  | Mild:Strong | 1 | 0.20134 | 0.2013 | 10.590 | 0.0116 | 0.0233 |
|  | Residuals | 8 | 0.15210 | 0.0190 |  |  |  |
| RLCS23 | Mild | 1 | 0.648 | 0.648 | 0.839 | 0.3865 | 0.5154 |
|  | Strong | 1 | 3.965 | 3.965 | 5.129 | 0.0533 | 0.0711 |
|  | Mild:Strong | 1 | 0.043 | 0.043 | 0.055 | 0.8198 | 0.8515 |
|  | Residuals | 8 | 6.184 | 0.773 |  |  |  |
| RLCS20 | Mild | 1 | 0.0858 | 0.0858 | 1.844 | 0.2116 | 0.3762 |
|  | Strong | 1 | 0.0477 | 0.0477 | 1.024 | 0.3413 | 0.3900 |
|  | Mild:Strong | 1 | 0.2935 | 0.2935 | 6.305 | 0.0363 | 0.0630 |
|  | Residuals | 8 | 0.3724 | 0.0466 |  |  |  |
| RLCS25 | Mild | 1 | 0.413 | 0.4126 | 0.892 | 0.3730 | 0.5154 |
|  | Strong | 1 | 1.125 | 1.1255 | 2.433 | 0.1570 | 0.1937 |
|  | Mild:Strong | 1 | 0.689 | 0.6888 | 1.489 | 0.2570 | 0.3164 |
|  | Residuals | 8 | 3.700 | 0.4625 |  |  |  |
| RLCS11 | Mild | 1 | 6.853 | 6.853 | 55.56 | 0.0001 | 0.0012 |
|  | Strong | 1 | 8.289 | 8.289 | 67.20 | 0.0000 | 0.0002 |
|  | Mild:Strong | 1 | 3.754 | 3.754 | 30.43 | 0.0006 | 0.0026 |
|  | Residuals | 8 | 0.987 | 0.123 |  |  |  |
| RLCS03 | Mild | 1 | 18.31 | 18.31 | 15.27 | 0.0045 | 0.0144 |
|  | Strong | 1 | 34.08 | 34.08 | 28.43 | 0.0007 | 0.0017 |
|  | Mild:Strong | 1 | 20.34 | 20.34 | 16.96 | 0.0033 | 0.0089 |
|  | Residuals | 8 | 9.59 | 1.20 |  |  |  |
| RLCS04 | Mild | 1 | 76.09 | 76.09 | 30.594 | 0.0006 | 0.0035 |
|  | Strong | 1 | 76.09 | 76.09 | 30.594 | 0.0006 | 0.0002 |
|  | Mild:Strong | 1 | 7.16 | 7.16 | 2.879 | 0.1282 | 0.1783 |
|  | Residuals | 8 | 19.90 | 2.49 |  |  |  |
| RLCS02 | Mild | 1 | 40.87 | 40.87 | 40.32 | 0.0002 | 0.0024 |
|  | Strong | 1 | 56.10 | 56.10 | 55.34 | 0.0001 | 0.0003 |
|  | Mild:Strong | 1 | 23.09 | 23.09 | 22.78 | 0.0014 | 0.0041 |
|  | Residuals | 8 | 8.11 | 1.01 |  |  |  |
| RLCS15 | Mild | 1 | 11.360 | 11.360 | 13.858 | 0.0058 | 0.0170 |
|  | Strong | 1 | 23.689 | 23.689 | 28.896 | 0.0007 | 0.0017 |
|  | Mild:Strong | 1 | 5.092 | 5.092 | 6.211 | 0.0374 | 0.0630 |
|  | Residuals | 8 | 6.558 | 0.820 |  |  |  |
| RLCS01 | Mild | 1 | 0.521 | 0.521 | 0.527 | 0.4884 | 0.6011 |
|  | Strong | 1 | 0.067 | 0.067 | 0.068 | 0.8004 | 0.8537 |
|  | Mild:Strong | 1 | 4.563 | 4.563 | 4.621 | 0.0638 | 0.1021 |
|  | Residuals | 8 | 7.900 | 0.987 |  |  |  |
| RLCS19 | Mild | 1 | 0.0037 | 0.0037 | 0.016 | 0.9033 | 0.9583 |
|  | Strong | 1 | 2.2963 | 2.2963 | 9.933 | 0.0161 | 0.0246 |
|  | Mild:Strong | 1 | 0.0067 | 0.0067 | 0.029 | 0.8700 | 0.8700 |
|  | Residuals | 8 | 1.6183 | 0.2312 |  |  |  |

Figure S1. Response categories and full data for of all 32 fungal isolates (based on colony extension rate) to recurrent heat pulse perturbations: synergy, antagony, and additive response.


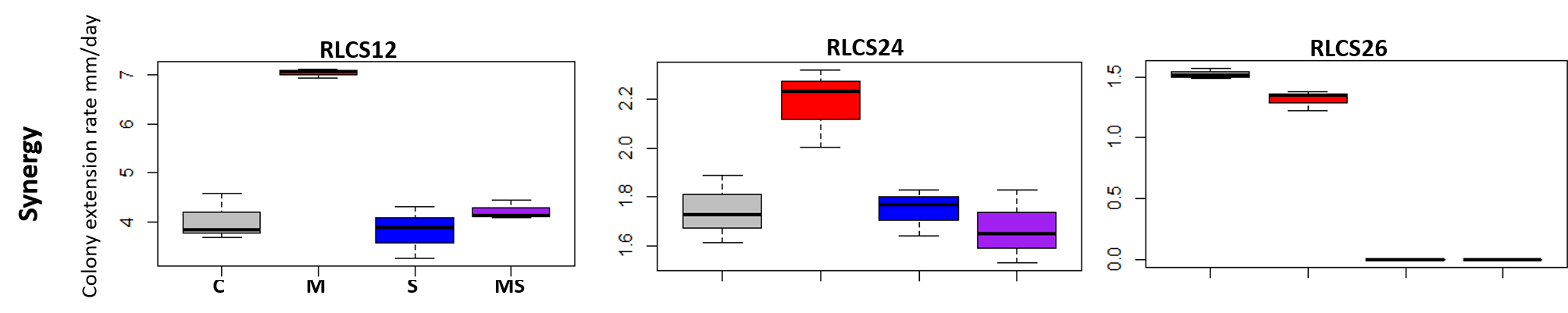

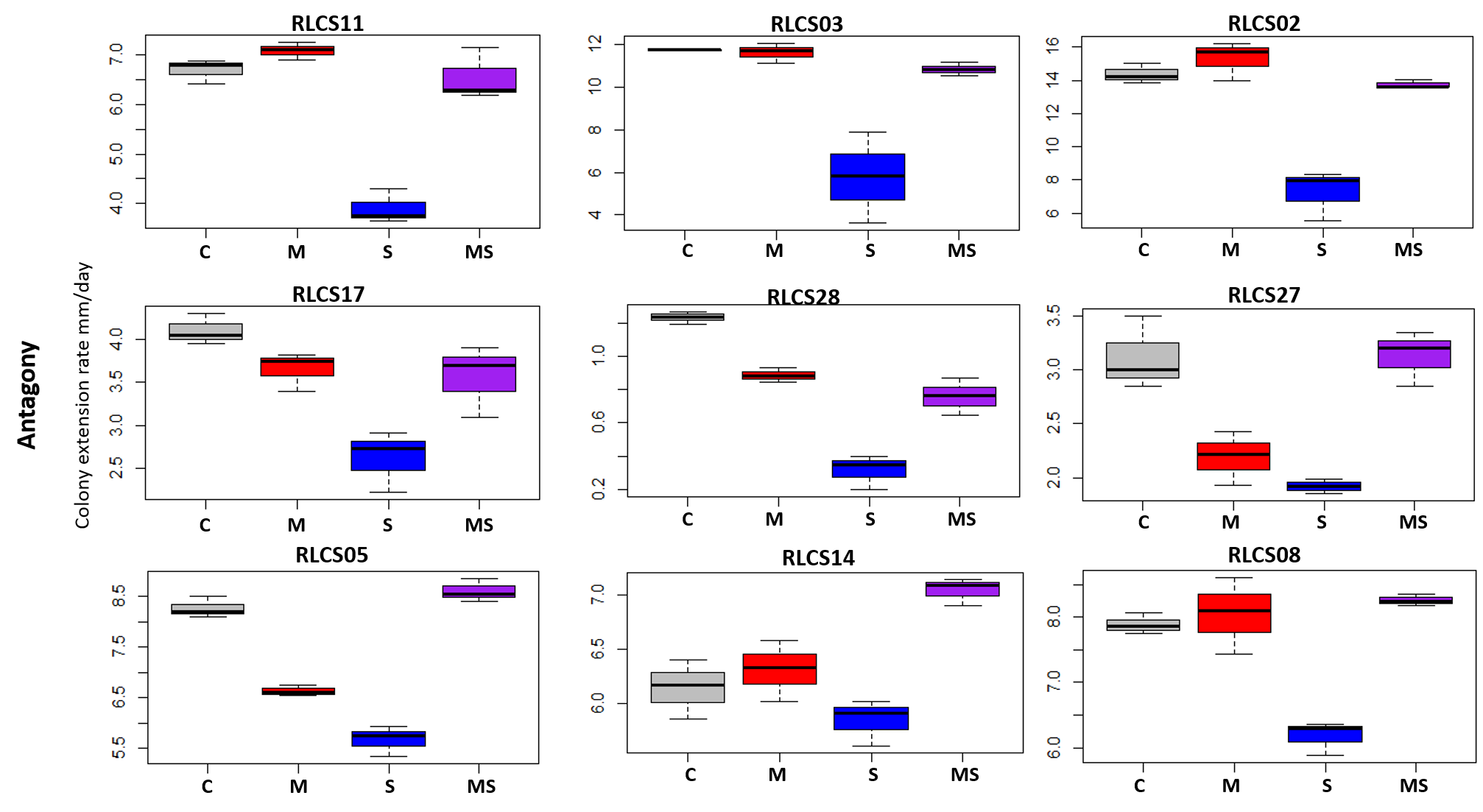

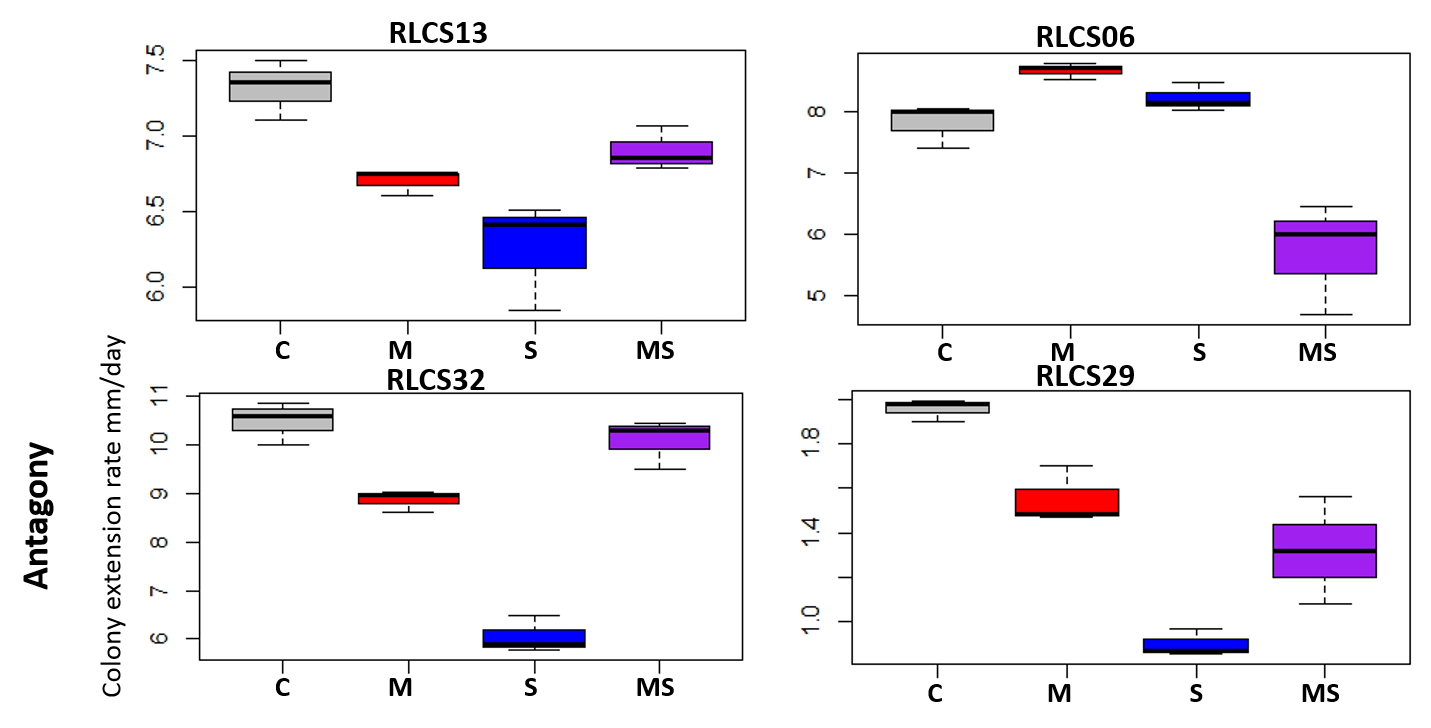

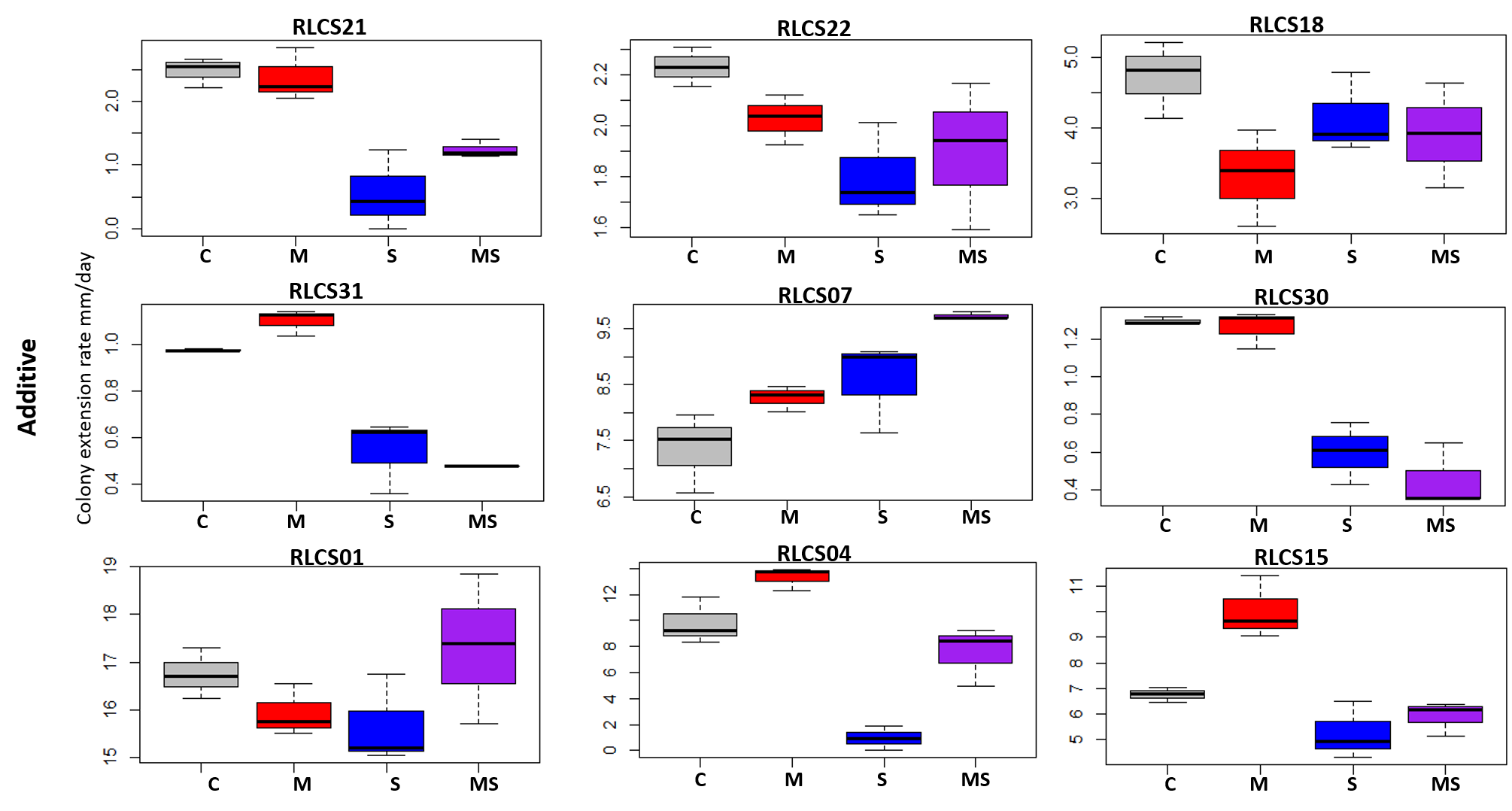

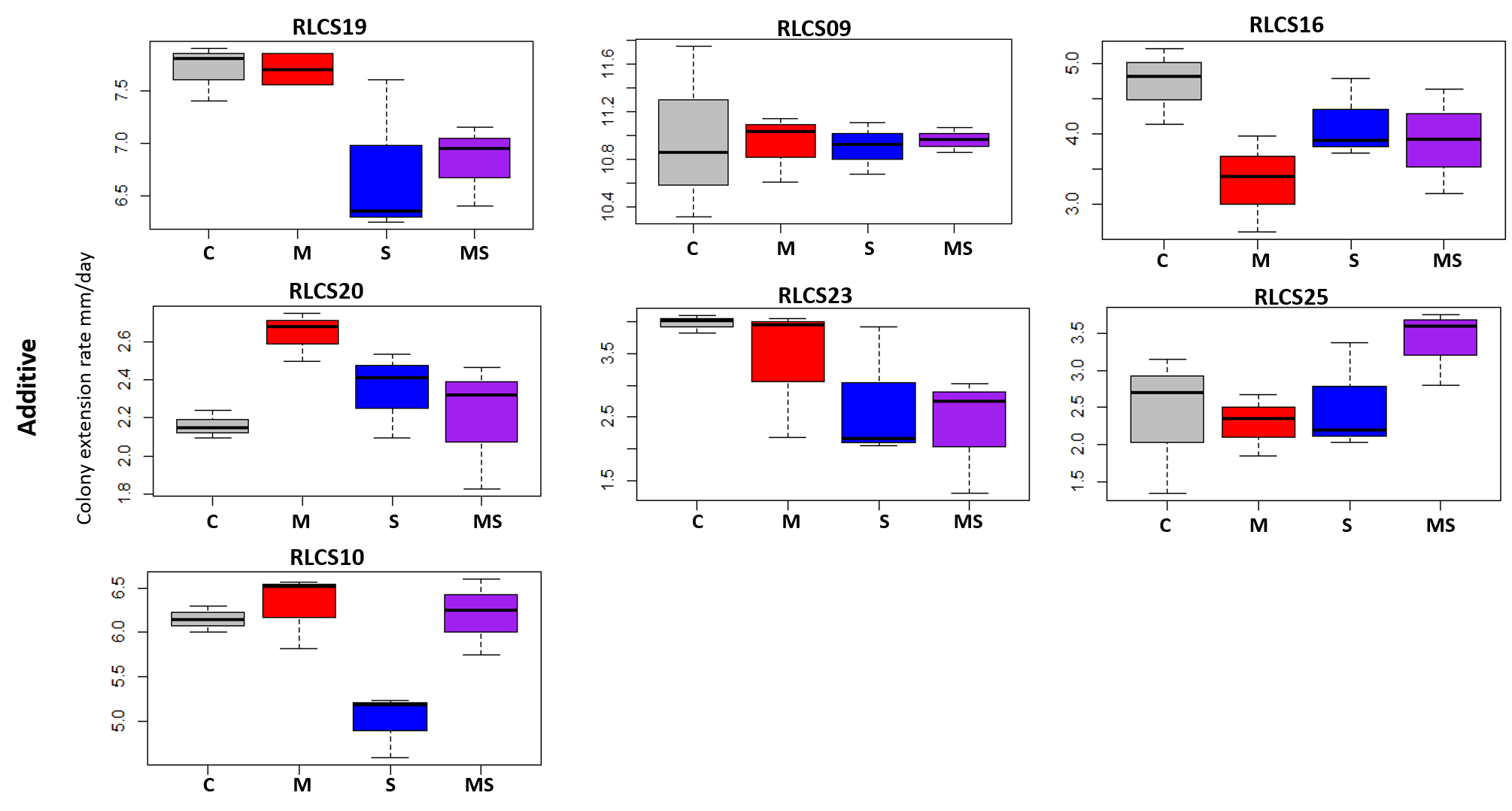

Supplement: Supplementary file 1 [file Data_Sheet_1.docx]
